# Platelet-activating factor (PAF) receptor as a promising target for cancer cell repopulation after radiotherapy

**DOI:** 10.1038/oncsis.2016.90

**Published:** 2017-01-30

**Authors:** I A da Silva-Jr, R Chammas, A P Lepique, S Jancar

**Affiliations:** 1 Departamento de Imunologia, Instituto de Ciências Biomédicas, Universidade de São Paulo, Sao Paulo, Brazil; 2 Faculdade de Medicina da Universidade de São Paulo, Instituto do Câncer do Estado de São Paulo, São Paulo, Brazil

## Abstract

A major drawback of radiotherapy is the accelerated growth of the surviving tumor cells. Radiotherapy generates a variety of lipids that bind to the receptor for platelet-activating factor, expressed by cells in the tumor microenvironment. In the present study, using the TC-1 tumor cell line, we found that irradiation induced a twofold increase in receptor expression and generated agonists of receptor. Irradiated cells induced a 20-fold increase in live TC-1 proliferation *in vitro*. Furthermore, subcutaneous co-injection of irradiated TC-1 cells with TC-1 expressing luciferase (TC-1 fluc^+^) markedly increased TC-1 fluc^+^ proliferation in a receptor-dependent way. Moreover we used a human carcinoma cell line not expressing the PAF receptor (KBM) and the same cell transfected with the receptor gene (KBP). Following co-injection of live KBP cells with irradiated KBM in RAG mice, the tumor growth was significantly increased compared with tumor formed following co-injection of live KBM with irradiated KBM. This tumor cell repopulation correlated with increased infiltration of tumor-promoting macrophages (CD206+). We propose that receptor represents a possible target for improving the efficacy of radiotherapy through inhibition of tumor repopulation.

## Introduction

Although radiotherapy is an effective way to control cancer locally, a major drawback of this treatment is the accelerated growth of surviving cells. This compensatory proliferation is an evolutionarily conserved process involved in tissue regeneration in lower animals, and is thought to occur with tumor cells treated with cytotoxic radiotherapy, as previously discussed.^1^ The alkyl- acyl-glycerophosphocholine (GPC), platelet-activating factor (PAF), binds to the PAF receptor. Irradiation generates reactive oxygen species,^2^ which act on membrane GPC to produce oxidized GPC, which also bind to the PAF receptor.^3^ Mass spectrometry revealed several different oxidized GPC molecules that bind to the PAF receptor.^4, 5^ Moreover, apoptotic and necrotic cells are phagocytosed by macrophages through scavenger receptors,^6^ and this process involves the association of CD36 with the PAF receptor.^7^ These macrophages acquire an anti-inflammatory phenotype, which is thought to explain the silent nature of this process, as it does not induce inflammation.^8^ Tumor irradiation may induce an anti-inflammatory microenvironment that favors tumor growth. Indeed, injection of apoptotic cells with a sub-tumorigenic dose of melanoma cells was found to promote tumor growth, and this was reversed by blocking PAF receptor signaling.^9^ The generation of anti-inflammatory macrophages and PAF receptor agonists in the tumor microenvironment may therefore represent possible mechanisms underlying radiotherapy failure. Furthermore, some tumor cells express the PAF receptor, and activation of this receptor with PAF increased the proliferation of human SKmel-23 melanoma cells.^10^ In the present study, we examined the effect of gamma radiation on the proliferation of PAF receptor-expressing tumor cells, and tumor cell repopulation.

## Results

### Irradiation increases the expression of the PAF receptor in TC-1 carcinoma cells and generates PAF receptor agonists

**Figure 1:**
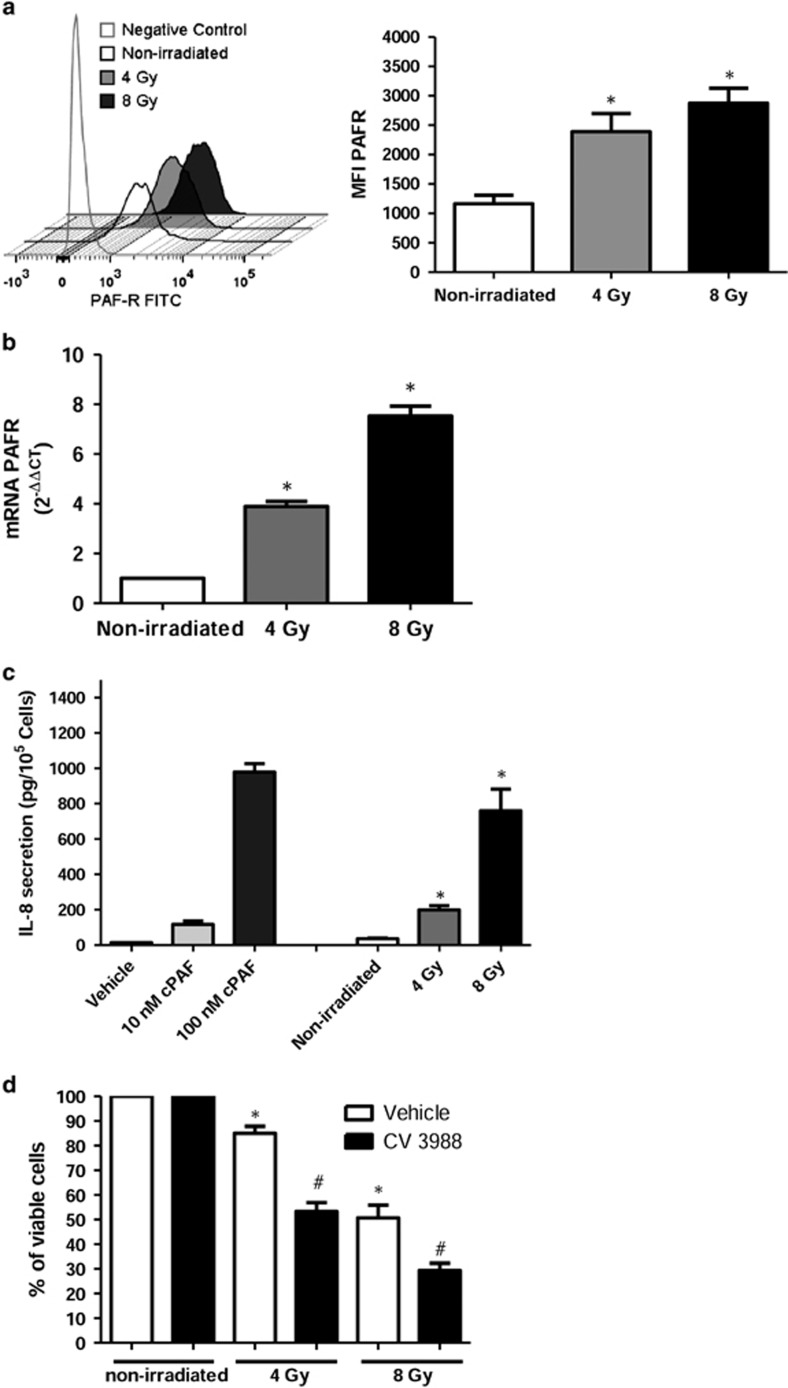
Radiation increases PAF receptor expression in TC-1 cells and induces generation of PAF receptor agonists. TC-1 tumor cells (5 × 10^4^) were cultured in RPMI medium containing 2% FBS and were exposed to different doses of ionizing radiation. (**a**) Flow cytometry analysis of membrane PAF receptor on TC-1 cells, filled and empty traces represent non-specific and specific binding for the PAF receptor, respectively. One representative experiment out of three is shown. Mean±s.e.m. of MFI values (*±0.05 irradiated vs non-irradiated); (**b**) RNA was extracted after 4 h of irradiation and PAF receptor mRNA levels were measured by real-time RT-PCR and represent the mean±s.e.m. of six experiments made in duplicate (*P<0.05 compared to non-irradiated control); (**c**) The TC-1 cells culture was collected after 1 h of irradiation and lipids extracts were assayed for PAF receptor agonistic activity, measured as IL-8 production by PAF receptor-expressing KBP cells. As control, PAF (10 and 100 nM) was added to KBP cells. Mean±s.e.m.* of six experiments made in duplicate<0.005, comparing irradiated with non-irradiated control; (**d**) Blocking of the PAF receptor with CV3988 potentiates irradiation-induced cell death. Mean±s.e.m. of four experiments made in duplicate (*P*<0.005 *comparing irradiated vs non-irradiated and #comparing CV3988 vs vehicle-treated).

The murine carcinoma cell line TC-1 was analyzed for the expression of the PAF receptor by flow cytometry analysis. The unfilled peaks represent specific antibody binding to the PAF receptor and, irradiation (4 and 8 Gy) significantly increased PAF receptor expression (Figure 1a). PAF receptor mRNA levels also increased with irradiation (2, 4 or 8 Gy), in a dose-dependent manner (Figure 1b). The lipid extract from the irradiated culture of TC-1 cells was then assayed for PAF receptor agonistic activity. This was achieved with an assay that uses KBM cells transfected with the PAF receptor (KBP cells), which secrete (interleukin) IL-8 in response to receptor activation. This is a very useful assay as it allows the detection of the collection of PAF receptor agonists generated by irradiation. Figure 1c shows that irradiation increased PAF receptor activation in a dose-dependent manner, compared with the non-irradiated cells. TC-1 cells irradiated with 8 Gy generated PAF receptor agonistic activity equivalent to 100 nM of PAF. Irradiation-induced dose-dependent apoptotic and necrotic cell death, shown in Figure 1d. Pre-treatment of TC-1 cells with the PAF receptor antagonist CV3988 before irradiation, significantly increased further cell death, supporting the protective effect of PAF on irradiated cells.

### PAF receptor mechanisms are implicated in increased tumor cell proliferation induced by radiation

**Figure 2:**
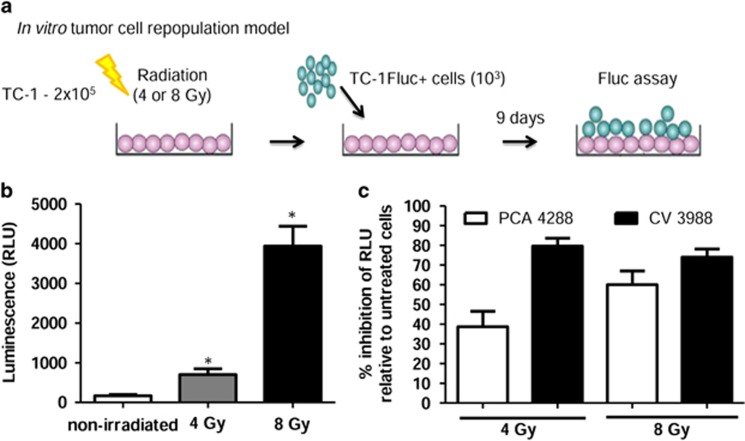
Irradiated cells exerts a 'feeder' effect on TC-1 cells via PAF receptor-dependent mechanism. (**a**) Schematic representation of experimental protocol: 2 × 10^5^ TC-1 cells were irradiated with 4 or 8 Gy and co-cultivated with 10^3^ TC-1 fluc^+^ cells for 9 days; (**b**) Relative luminescence units (RLU) in TC-1 cells irradiated (4 or 8 Gy); (**c**) % inhibition of RLU in cells pre-treated with PAF receptor antagonists PCA4248 or CV3988, both at 10 μM, relative to untreated cells. Data represent the mean±s.e.m. of six experiments done in duplicates. The difference between each of the irradiated groups (4 and 8 Gy) and controls (0 Gy) and between treated or not with PAF receptor antagonists was statistically significant (*P<0.05).

**Figure 3:**
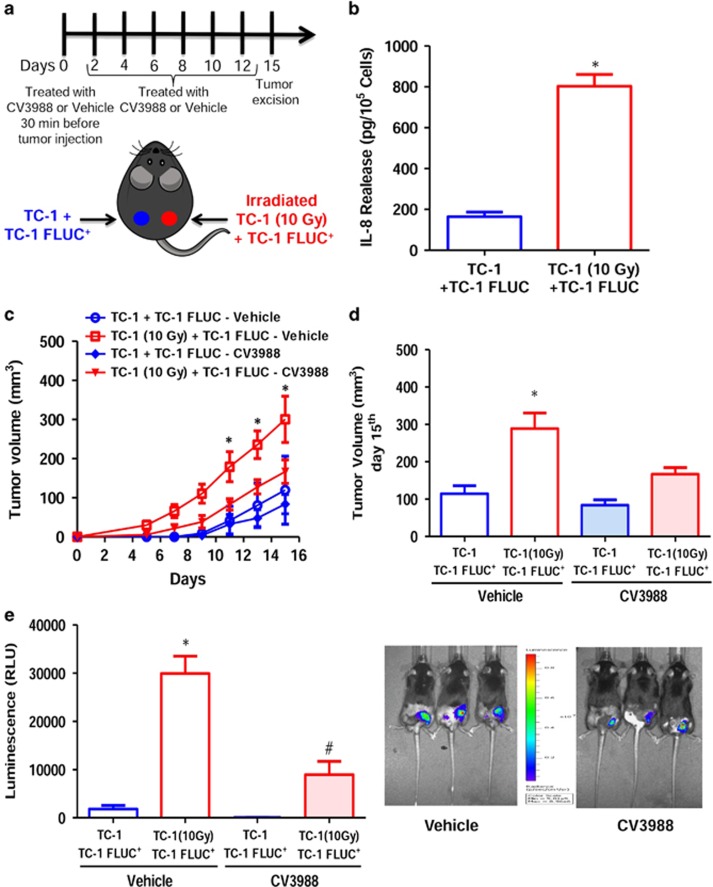
TC-1 tumor cells growth *in vivo* is increased when mixed with irradiated cells. (**a**) Schematic representation of the protocol used in these experiments. Irradiated (10 Gy) or control TC-1 cells (5 × 10^5^) were injected subcutaneously into the shaved back of C57BL6 mice together with non-irradiated 10^3^ TC-1-Fluc. These animals were treated with intraperitoneal injection of the PAF receptor antagonist (CV3988) in a dose of 10 mg/kg or vehicle (PBS) every 2 days; (**b**) PAF receptor agonistic activity in lipid extracts from tumor mass after 1 h of 10 Gy, measured as IL-8 production by PAF receptor-transfected KBP cell (*n*=6). (**c**) Effect of dying TC-1 on TC-1 fluc^+^ tumor cell growth *in vivo*, shown as tumor volume (mm^3^) followed during 15 days in the presence or absence of CV3988; (**d**) focus on the differences in tumor volume between groups at day 15; (**e**) Left, quantification of bioluminescent signals from tumors. Right, representative bioluminescent images of mice treated (right) or non-treated (left) with the PAF receptor antagonist (*n*=7 animals per group).

The ability of TC-1 cells to stimulate the growth of non-irradiated TC-1 cells was tested according to the protocol illustrated in Figure 2a. A 'feeder layer' of TC-1 cells (2 × 10^5^) was left to rest in culture for 24 h before irradiation with 4 or 8 Gy. A smaller number (10^3^ cells) of TC-1 fluc^+^ was then added to the cultures and a luciferase assay was performed following a 9  day incubation. Luminescence was linear with TC-1 fluc^+^ cell number (Supplementary Figure 1). The presence of irradiated feeder cells significantly potentiated TC-1 proliferation, as shown in Figure 2b. When TC-1 fluc^+^ cells were treated with the PAF receptor antagonists before irradiation (4 Gy), PCA4288 caused 40% reduction in cell proliferation and CV3988 caused 80% reduction, when compared with the control, vehicle-treated cells. Following treatment with a higher dose of irradiation (8 Gy), both PAF receptor antagonists were similarly effective in their reduction of cell proliferation (Figure 2c). To evaluate whether these observations also occur *in vivo,* TC-1 cells (2 × 10^5^) were irradiated with 10 Gy and injected subcutaneously together with a smaller number (1 × 10^4^) of non-irradiated TC-1 fluc^+^ cells, in animals with or without PAF receptor antagonist (CV3988) incubation, as illustrated in Figure 3a. As shown in Figure 3c, when live TC-1 cells were mixed with irradiated cells, the tumor volume was significantly larger than those mixed with viable cells. The treatment with the PAF receptor antagonist reduced the growth of the tumor formed by the mixture of live with irradiated cells (Figure 3d). Consistent with the *in vitro* observation (Figure 2), the irradiated cells promoted increased proliferation of the TC-1-Fluc viable cells at day 15 (Figure 3e), and the treatment with CV3988 diminished this cell proliferation and tumor size. Moreover, the lipid extracts from these tumors showed increased level of PAF receptor agonistic activity, measured as IL-8 production and subsequent activation for the PAF receptor in KBP cells (Figure 3b). Taken together, the *in vitro* results indicate that PAF-like molecules are produced by radiation, enhancing the proliferation of live TC-1 fluc^+^ cells. The *in vivo* results indicate that the presence of irradiated cells stimulates the growth of live tumor cells and a release of PAF receptor agonists in the tumors.

PGE_2_ can mediate tumor growth, induced by apoptotic cells through EP_2_ signaling.^9^ We have previously observed that the high levels of PGE_2_ present in Ehrlich ascites tumor were significantly reduced in mice treated with PAF receptor antagonists, suggesting that the production of PGE_2_ is dependent on PAF receptor ligands present in the ascites.^11^ Therefore, we stimulated TC-1 cells with cPAF (100 μm) and found that it induced PGE_2_ production. Moreover, irradiation (8 Gy) of TC-1 cells also induced PGE_2_ production and it was significantly reduced by the PAF receptor antagonist, CV3988 (Supplementary Figure 2).

### PAF receptor and radiation-induced tumor cell repopulation

**Figure 4:**
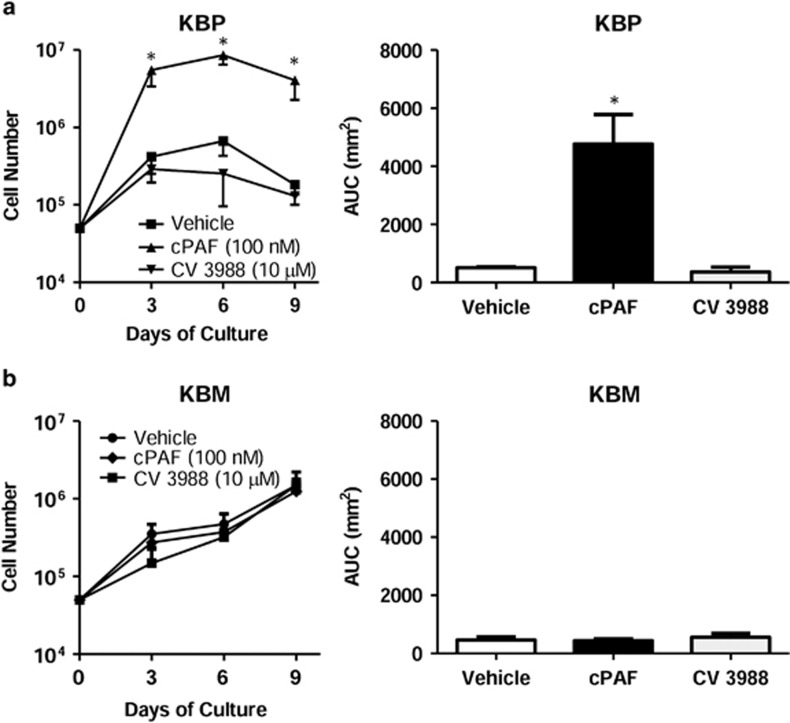
PAF receptor agonist (cPAF) augments tumor cell proliferation *in vitro*. (**a**) KBP cells and (**b**) KBM cells treated with either vehicle or cPAF (100 nM) or CV3988 (10 μM). The cell growth was evaluated every 3 days until 9 days. The data are represented as mean±s.e.m. of cell number (left panel) or area under curve (right panel) from six independent experiments. Significant difference was observed between cells treated with cPAF or vehicle (*P<0.05).

**Figure 5:**
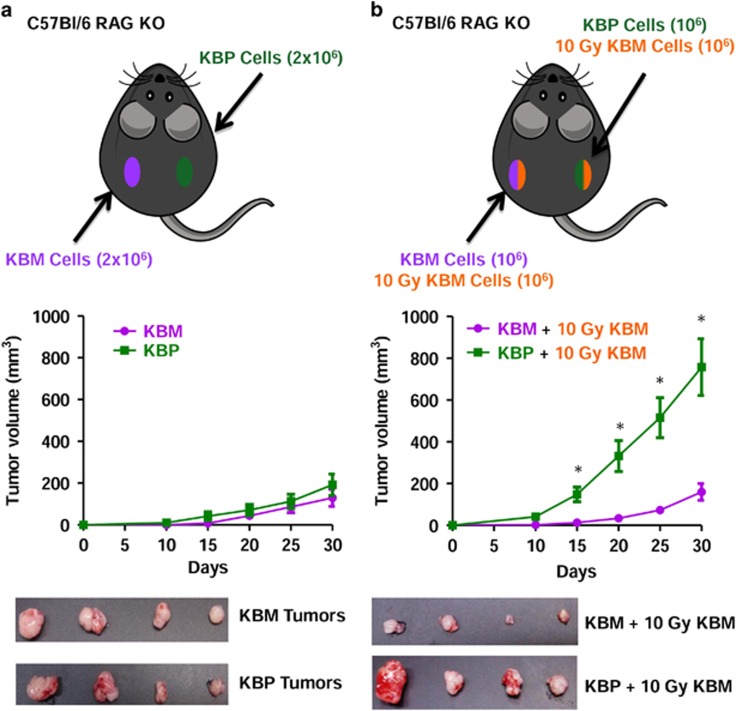
Repopulation is dependent on the expression of PAF receptor in the tumor cells. (**a**) Schematic representation of the experimental protocol. KBM or KBP cells (2 × 10^6^) were injected subcutaneously on the dorsal hind flank skin of C57BL/6 RAG KO. (**b**) Mix of 1:1 live and irradiated KBP/KBM or KBM/KBM cells in a total of 2 × 10 cells were injected subcutaneously in the right and left flanks of mice, respectively. *Significant differences (*P*<0.05) were observed for KBP compared with KBM tumors. The tumors were measured with calipers every 5 days for 30 days. The data are represented as mean±s.e.m. of tumor volume (*n*=12 animals per group).

To investigate if PAF receptor-mediated mechanisms are involved in tumor repopulation after radiotherapy, human carcinoma cells (KBM) that do not express PAF receptor, and were irradiated, were co-injected with the same cells transfected with the PAF receptor (KBP). In this case, cells were injected into C57BL6 Rag KO mice to avoid rejection of the human tumor cells. This model has the advantage of avoiding the interference of adaptive immunity on tumor growth, allowing the role of innate immunity on tumor repopulation to be determined. Figure 4a shows that treatment of KBP cells with the PAF receptor agonist cPAF (100 nM) resulted in increased *in vitro* proliferation, whereas the KBM cells did not proliferate in response to cPAF treatment (Figure 4b). When these cells were injected subcutaneously into RAG KO mice, both tumors grew slowly and after 30 days, KBM and KBP developed into tumors of similar volume (Figure 5a). However, when the KBP was mixed with irradiated (10 Gy) KBM cells, the tumor grew rapidly and was significantly larger than the tumor that resulted from the mixture of KBM cells with irradiated KBM cells (Figure 5b).

### The PAF receptor and tumor macrophages

**Figure 6:**
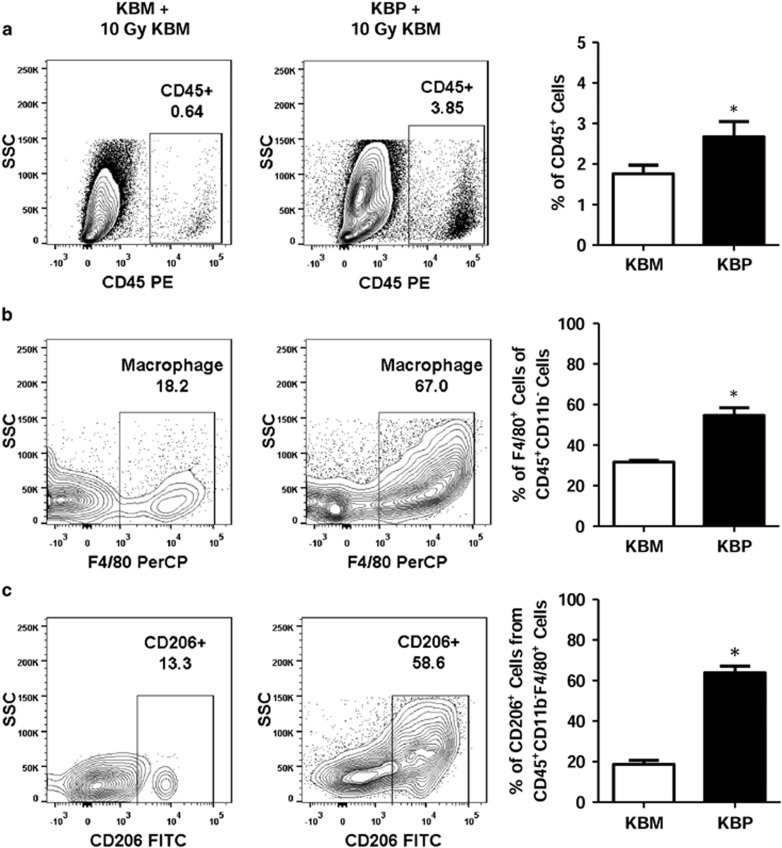
KBP tumor repopulation is accompanied by increased regulatory macrophages (CD206+) infiltration in the tumor mass. Macrophage analyses from tumors derived from KBM cells (left) or KBP cells (right) mixed with irradiated KBM cells (10 Gy). The tumor cells were isolated by enzymatic digestion and stained for flow cytometry analysis. Results are shown as representative plots and as means±s.e.m. (*n*=12 animals per group) of (**a**) CD45+ population (leucocytes) (**b**) F4/80+ population (macrophages) and (**c**) CD206+ population of F4/80+ cells (M2 macrophages). *P<0.05 for KBM compared with KBP tumors.

Cells that do not survive radiotherapy are cleared from the tumor environment by tumor macrophages, in a process that is dependent on the PAF receptor. During this process, macrophages acquire the anti-inflammatory phenotype M2,^7^ which has been shown to stimulate tumor angiogenesis and tumor growth.^12^ We investigated the tumor macrophages phenotype, using the KBM or KBP cells mixed with irradiated KBM cells and injected subcutaneously. After 30 days, the tumors were excised and examined for leukocytes infiltration by flow cytometry. For flow cytometry analysis, we first made a gate on the leukocyte population (CD45+) and within this population analyzed the macrophage population (F4/80+). The latter was then analyzed using the M2 marker, CD206. A clear relationship between the presence of the PAF receptor in the KBP tumor cells and increased infiltration of leukocytes was observed (Figure 6a). Moreover, the frequency of tumor macrophages was higher with the KBP cells than the KBM (Figure 6b). In addition, the KBP tumors compared with KBM showed a higher influx of cells expressing CD206, which is a marker for tumor-promoting M2 macrophages (Figure 6c).

## Discussion

Our results show that irradiation increased PAF receptor expression in the TC-1 tumor cell line and generated molecules that activate the PAF receptor. Irradiated cells exerted a feeder effect on live TC-1 cells *in vitro* by stimulating cell proliferation. Irradiated TC-1 injected subcutaneously in mice together with TC-1 fluc^+^, markedly increased tumor growth. In an *in vivo* repopulation experiment, when KBP cells were co-injected with irradiated KBM cells, the tumors were much larger than the ones formed by co-injection of KBM cells with irradiated KBM cells. The repopulation phenomenon correlated with increased frequency of M2 macrophages. A possible interpretation of these results is that irradiation induces cell death and generates molecules that bind to the PAF receptor. When injected *in vivo*, the irradiated cells may create a 'niche' in which the live tumor cells would have an accelerated growth, dependent on activation of the PAF receptor in tumor cells as well as tumor macrophages. Dying cells express moieties in their membranes that bind to PAF receptor^13^ and are cleared by macrophages by mechanisms that involve the scavenger receptor CD36 and PAF receptor.^7^

In the first part of this study, we show that TC-1 cells express the PAF receptor and treatment with PAF receptor agonist stimulated tumor cell proliferation. In various types of cells, PAF treatment induces phosphatidylinositol hydrolysis, arachidonic acid release and MAP kinases activation that trigger proliferative signals in tumor cells.^14^ Expression of the PAF receptor has been reported in pro-myelocytic leukemia cells,^15^ colorectal carcinoma,^16^ human esophageal cancer cells^17^ and human breast carcinoma.^18^ Moreover, PAF is thought to have an important role in tumor growth.^19, 20^

The present study describes that gamma radiation of TC-1 cells generated significant levels of PAF receptor agonistic activity, assayed using a KBP cell, IL-8 releasing assay. This assay allows for detection of all ligands of the PAF receptor, and not only the agonist PAF present. Recently it was shown that chemotherapeutic agents and radiotherapy induce production of PAF-like molecules^21, 22^ and a plethora of oxidized phospholipids that can bind to the PAF receptor. Therefore, despite cell death induced by these treatments, they also induce generation of PAF-like molecules, which may promote proliferation of treatment-resistant cells, by activating the PAF receptor expression in these cells. This study reported that irradiation induced PAF receptor ligands and promoted overexpression of the PAF receptor in tumor cells, which was found correlate with tumor cell repopulation. Blocking the PAF receptor with the antagonist CV3938 or PCA4280 before irradiation further decreased TC-1 cell viability, suggesting that PAF receptor agonists protect tumor cells from death induced by radiotherapy. We also observed that blocking of PAF receptor abolished the effect of irradiation-induced TC-1 proliferation. To our knowledge, the role of these signaling events in TC-1 tumor repopulation has not been previously described.

Early work from REVESZ^23^ described that a mixture of tumor cells killed by radiation with viable cells led to increased proliferation of the latter and that this was dependent on diffusion of a growth-stimulating metabolite. Our results identify the growth-stimulating metabolite as lipid molecules that bind to the PAF receptor.

Several studies have attempted to understand the molecular mechanisms of tumor repopulation after cytotoxic therapy. Huang *et al.* 2011 showed that activated caspase-3 in tumor cells undergoing apoptosis has a major role in repopulation after radiotherapy by inducing PGE_2_ production. PGE_2_ has a crucial role in tumor cell proliferation and can be essential for tumor repopulation. In a previous study, we showed that the combination of chemotherapy (Dacarbazine) with the PAF receptor antagonist (WEB2170) reduced the proportion of caspase-3 and COX-2-positive cells within the tumor.^24^ Thus, it is possible that 'PAF-like' molecules may potentiate tumor cell growth, either by attenuating the cytotoxic effects of radiotherapy or stimulating tumor cell proliferation.

It is interesting to note the activated caspase-3 leads to activation of cPLA2,^25^ which acts on phosphatidylcholine generating one molecule of arachidonic acid and lysophosphorycholine. Although arachidonic is a substrate for cyclooxygenases leading to prostanoid synthesis, upon acetylation, lysophosphorycholine converts into PAF. Therefore, the same reaction that stimulates PAF synthesis can stimulate PGE_2_ production, exerting a positive feedback loop in repopulation phenomenon.

We obtained direct evidence for a stimulatory role of PAF receptor-induced proliferation. Stimulation of KBP cells, but not of KBM cells, with carbamyl-PAF, which is not inactivated by serum acetyl-hydrolase, induced an increase in cell proliferation. These results are in accordance with the work of Bussolati *et al.*^14^ showing that endogenous or exogenous administration of PAF induces an increase in cell proliferation in breast cancer cells that express the PAF receptor without affecting proliferation in PAF receptor-negative breast cancer.

In addition, we found that injection of a large number of irradiated KBM cells mixed with KBP cells resulted in tumor growth, whereas this was not observed when KBM cells were mixed with irradiated KBM cells. This strengthens the idea that the presence of dying cells in the tumor microenvironment stimulates tumor growth.

Our results show a clear relationship between the presence of the PAF receptor in the tumor cells and increased infiltration of leukocytes, mainly composed of macrophages expressing CD206, which is a marker for tumor-promoting M2 macrophages. Although macrophages have high plasticity and can exhibit a wide range of profiles, for didactic purposes they have been grouped in defined phenotypes.^26^

These data suggest that PAF receptor-dependent mechanisms modify the tumor microenvironment, including the phenotype of tumor macrophages. Interactions between tumor and stromal cells can occur via cell–cell interactions, or by cytokine- or chemokine-mediated signaling.^27^ We propose here that these interactions also involve PAF receptor-mediated signaling favoring the tumor progression by suppressing macrophage functions.

Finally, these findings imply a dual role for the PAF receptor in tumor repopulation induced by radiotherapy. 'PAF-like' molecules generated by radiotherapy by their action on tumor cells protects them for radiation-induced cell death and by acting on macrophages, stimulates the tumor growth through immunosuppression. Therefore, association of radiotherapy with the PAF receptor antagonist represents a promising strategy for improving the efficacy of radiotherapy.

## Material and methods

### Cell lines and culture conditions

TC-1 cells, an established murine SCC line, were kindly donated by Dr TC Wu (John Hopkins, Baltimore). TC-1 fluc^+^ cells were obtained by transfection with a vector containing the hygromycin B resistance gene and the firefly luciferase gene (Fluc). Cells were maintained in Rosewell Park Memorial Institute (RPMI) medium, supplemented with 10% fetal calf serum, hygromycin B (100 μg/ml), neomicin (400 μg/ml), penicillin (100 units/ml) and streptomycin (100 μg/ml). In addition, we obtained a PAF receptor-negative human epithelial cell line (KBM) and PAF receptor-positive (KBP) cells from Dr JB Travers (Department of Dermatology, Indiana University School of Medicine, Indianapolis, IN, USA). These cell lines were cultured in DMEM (Dulbecco's modified Eagle's medium, GIBCO, Waltham, MA, USA) supplemented with 10% fetal calf serum, penicillin (100 units/ml) and streptomycin (100 μg/ml). Cells have been regularly tested for Mycoplasma and were free of this contamination. All cell cultures were incubated at 37 °C under a humidified atmosphere of air containing 5% CO_2_. A growth curve of control and PAF receptor agonist treated (100 nM cPAF) KBM and KBP cells was performed.

### Animal model

The experiments were performed using 6- to 8-week-old male C57BL6/C or C57BL6/C RAG knockout mice from the animal breeding facility in the Department of Immunology, São Paulo University. The animal facility and subsequent experiments were approved according to the Brazilian Animal Welfare Regulations (National Council for Animal Experimentation Control—CONCEA, protocol number 130/2015). The animal rooms provided daylight plus a 12 h light–dark electric cycle and a constant temperature of 26 °C and relative humidity of 50–60%. The mice were fed a laboratory animal diet and sterile water *ad libitum*. Tumor cells were injected subcutaneously as single-cell suspensions in phosphate-buffered saline (PBS; 2 × 10^6^ cells in 100 μl) into the right and left hind leg of the mice. Some tumor cells were irradiated before injection, and these treatments are indicated in the appropriate Figures as well as the number of animals per group. Tumor volume was calculated using the equation: *v*=(*d*_1_ × *d*_2_)^2^ × 0.6, where *V* is the tumor volume, *d*_1_ is the largest diameter of the tumor and *d*_2_ is the smallest diameter of the tumor. None of the animals injected with tumor cells was excluded from the analysis. A method of randomization was not used to allocate the experimental groups. Allocation of the animals and assessment of the outcome were done without blinding.

### *In vitro* irradiation of TC-1 tumor cells

TC-1 cell lines were grown on 10 cm dishes to 80–90% confluence, and washed three times with pre-warmed (37 °C) PBS and then cultured in RPMI medium containing 2% fetal bovine serum. Tumor cells were irradiated with multiple doses of gamma radiation (Gy) and incubated for 1 h or 6 h time points, as detailed in the Figure legends. Cell irradiation studies were conducted using an IBL 136 cell and animal gamma radiator machine (Compagnie Oris Industrie, Gif-sur-Yvette, France). Settings for the machine were as follows: *d*=33 cm, and dose rate of 251.7 cGy/min. In some experiments, the PAF receptor antagonist (CV3988; Enzo Life Sciences, Farmingdale, NY, USA) or the vehicle control dimethyl sulfoxide (0.5%) were pre-incubated for 30 min before irradiation. After 48 h, the cells were collected and counted, and cell viability examined by the trypan blue exclusion test. The expression of the PAF receptor in carcinoma tumor cell lines was assessed by flow cytometry and real-time reverse transcriptase PCR.

### Flow cytometry for PAF receptor expression

Irradiated TC-1 cells and non-irradiated TC-1 cells were incubated for 48 h. The cell culture medium was then replaced with ice-cold PBS and the cells were removed with a rubber policeman cell scraper and harvested by centrifugation at 250 *g* for 5 min. Following centrifugation, the cell pellet was washed and re-suspended in staining buffer (PBS, fetal calf serum 1%, sodium azide 0.1%), containing the anti-PAF receptor primary antibody (1:100 dilution in staining buffer; Cayman Chemical, Ann Arbor, MI, USA). Following a 30 min incubation, the cells were washed and re-suspended in staining buffer containing Alexa Fluor 647-goat anti-rabbit IgG secondary antibody (1:100 dilution in staining buffer; Invitrogen-Life Technologies, Carlsbad, CA, USA). Cells incubated with secondary antibody only were used to control for background fluorescence. The expression of the PAF receptor was analyzed by flow cytometry using BDFACS-Canto II (BD Biosciences, San Jose, CA, USA) and FlowJo Version 5.0 software (TreeStar, Ashland, OR, USA). During data acquisition, the doublets were excluded using gates in FSC-A vs FSC-H. The autofluorescence of TC-1 cells was removed at the beginning of the analysis.

### RNA analysis for PAF receptor expression

PAF receptor expression was analyzed by reverse transcriptase PCR using RNA obtained from TC-1 cells, collected 6 h post irradiation. Total RNA was isolated using TRIzol (Life Technologies). For real-time reverse transcriptase PCR, cDNA was synthesized using the RevertAid First Strand cDNA Synthesis Kit (Fermentas Life Sciences, Ontario, CA, USA), according to the manufacturer's instructions. PCR-master mix (Power SyBr Green, Applied Biosystems, Warrington, UK) containing the specific primers was then added. Human PAF receptor forward primer: GGG GAC CCC CAT CTG CCTCA and reverse GCG GGC AAA GAC CCA CAG CA; GAPDH forward primer: GAG TCA ACG GAT TTG GTC GT and reverse primer: TTG ATT TTG GAG GGA TCT CG. Real-time PCR was performed using the Mx3005PTM Real-Time PCR System. Relative gene expression was calculated using the 2^−ΔΔC^_T_ method, as previously described.^28^ Results are presented as a fold increase relative to non-irradiated cells.

### Measurement of PAF receptor ligands after irradiation

TC-1 cells (10^7^ ml) were plated in 10 cm dishes and incubated overnight in DMEM supplemented with 10% fetal calf serum medium. Following incubation, the culture medium was replaced with 2 ml of pre-warmed (37 °C) Hanks Balanced Salt Solution, supplemented with 10 mg/ml fatty acid-free bovine serum albumin and 2 μM Pefabloc, a serine hydrolase inhibitor (Sigma-Aldrich, St Louis, MO, USA). The cells were then irradiated with 2–8 Gy following a 1 h incubation, the culture was quenched by addition of 2 ml of ice-cold methanol followed by methylene chloride, and lipids were extracted, as previously described.^29, 30, 31^ The ability of irradiation to produce PAF receptor agonists was also tested *in vivo*, using TC-1 tumors that were implanted subcutaneously into the left hind flanks of C57BL6/c animals. Mice were anesthetized 1 h after irradiation and tumors dissected, weighed and lipids were extracted. The presence of PAF receptor ligands in lipid extracts was determined by the ability of these extracts to induce IL-8 production in KBP cells, as previously described.^32^ In brief, 2 × 10^−5^ KBP cells were plated in 12-well plates and cultured overnight in DMEM containing 10% (v/v) fetal bovine serum. The cells were then washed with PBS, and incubated with fetal bovine serum-free DMEM. The cells were stimulated with the lipid extracts from irradiated TC-1 cells and after 6 h, the supernatants were collected for IL-8 measurement by ELISA. The concentration of IL-8 induced by the lipid extracts was compared with that induced by the stable PAF receptor agonist cPAF (1-hexadecyl-2-N-methylcarbamoyl glycerolphospho-choline). The concentration of IL-8 in the supernatants was measured using BD OptEIA ELISA sets (BD Biosciences, San Diego, CA, USA), according to the manufacturer's instructions.

### Bioluminescence imaging

The TC-1 fluc^+^ cells were imaged in the IVIS200 instrument (Caliper Life Sciences, Hopkinton, MA, USA). To monitor growth of fluc^+^-labeled cells *in vitro*, ~1000 cells were mixed together with 2 × 10^5^ unlabeled cells, immediately after irradiation. In some experiments, blockage of the PAF receptor was performed using the chemically unrelated antagonists CV3988 or PCA4288 (Tocris Biosciences, Ellisville, MO, USA), which were added to TC-1 culture 30 min prior to irradiation. The growth of TC-1 fluc^+^ cells was monitored following 9 days of culture, by adding Rosewell Park Memorial Institute medium with 0.15 mg/ml D-luciferin (Caliper Life Sciences). Cells were imaged 2 min after the addition of d-luciferin. The growth of fluc^+^-labeled tumor cells *in vivo* was monitored by non-invasive bioluminescence imaging. Mice were imaged following intraperitoneal injection with 150 mg/kg of D-luciferin in 200 μl of PBS, and then anesthetized with a continuous flow of isofluorane. Imaging of the mice was carried out 10 min later. Acquired images were analyzed following the manufacturer's instructions.

### Flow cytometry analyses of macrophages from KBM/KBP tumors

For FACS analyses, tumors were harvested following animal euthanasia, and digested with 100 μg/ml collagenase with constant stirring at 37 °C for 45 min. Cell pellets were re-suspended in FACS buffer (PBS containing 0.1% sodium azide and 1% fetal bovine serum) and stained with fluorescent-conjugated monoclonal antibodies: Pacific Blue anti-mouse CD45 Antibody (30-F11); PE-Cy5.5 conjugated F4/80 (clone BM8); FITC anti-mouse CD206 (C068C2), all purchased from eBiosciences (San Diego, CA, USA). The cell suspension was filtered through a 70 μm mesh and washed twice prior to analyses by flow cytometry, as described above. Results were collected for 100 000 cells and analyzed using the FlowJo software (TreeStar). F4/80+ populations were determined on CD45+ gated cells.

### ELISA measurement of PGE_2_

To measure PGE_2_ secretion by TC-1 cells, 10^6^ cells/well were plated in 10 cm dishes and incubated overnight. The cells were treated with the COX inhibitor, indomethacin (Sigma-Aldrich) or the PAF receptor antagonist (CV3988), for 30 min prior gamma radiation treatment. The supernatants from the cells were collected 1 h after cell irradiation and PGE_2_ levels in the supernatants were measured by ELISA (Cayman Chemical, Minneapolis, MN, USA).

### Statistical analysis

Data are represented as mean±s.d. unless otherwise indicated and were analyzed using the Prism 5.0 statistical program (GraphPad Software, San Diego, CA, USA). No sample or animal exclusion criterion was used. Comparisons among groups were performed by analysis of variance followed by the Bonferroni multiple comparison test. A two-sided *P*<0.05 was considered statistically significant. Each experiment was repeated at least three times.
