# Supplementary material for: Platelet-activating factor (PAF) receptor as a promising target for cancer cell repopulation after radiotherapy
Source: Oncogenesis. 2017 Jan 30;6(1):e296–. doi: 10.1038/oncsis.2016.90 (PMC5294253; doi:10.1038/oncsis.2016.90)
Supplement: Supplementary Information [file oncsis201690x1.docx]

**Supplementary Figure Legend**

**Supplementary Figure 1. Linearity of measured bioluminescence vs TC-1 fluc+ cell numbers.** TC-1 fluc+ cells were plated in 96 well dishes in duplicates in different numbers. They were then imaged by use of the IVIS200 imaging system. Top panel, luminescence plotted against cell number (R2 = 0.98, two-tailed ANOVA analysis). Error bars represent standard error of the mean (SEM) of 3 experiments. Lower panel, an example of fluorescence image of the plated TC-1 fluc+ cells.

**Supplementary Figure 2. Irradiation induces PGE2 production.** TC-1 cells culture were harvested after 1h of irradiation (8 Gy) and PGE2 concentration was measured. As control, PAF (100 nM) and indomethacin were added to TC-1 cells. Mean ± SEM (p < 0.005 *comparing irradiated or PAF vs non-irradiated and #comparing CV 3988 vs irradiated)
