# Supplementary figures and images for: Platelet-activating factor (PAF) receptor as a promising target for cancer cell repopulation after radiotherapy
Source: Oncogenesis. 2017 Jan 30;6(1):e296–. doi: 10.1038/oncsis.2016.90 (PMC5294253; doi:10.1038/oncsis.2016.90)

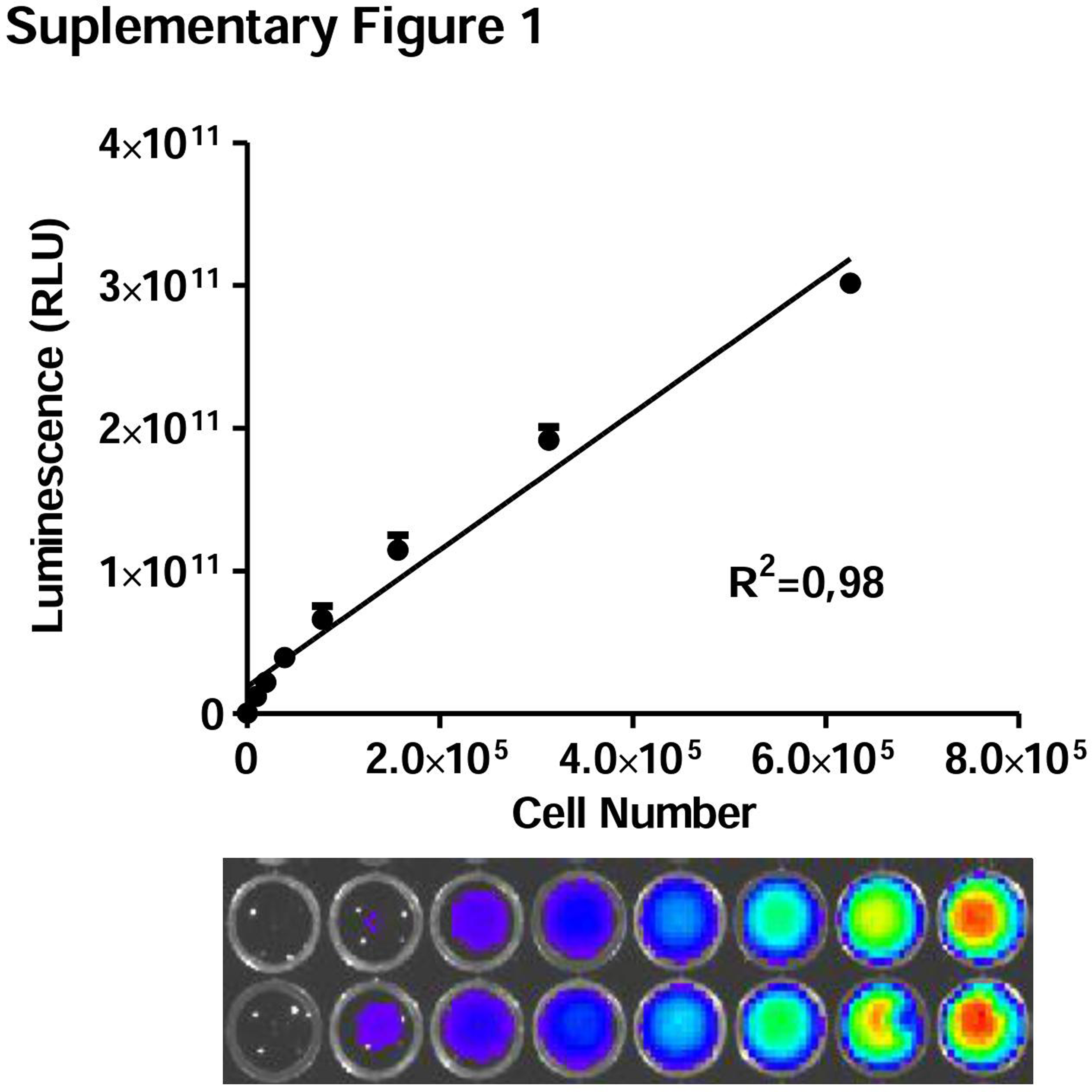

Supplement: Supplementary Figure 1 [file oncsis201690x2.tif]

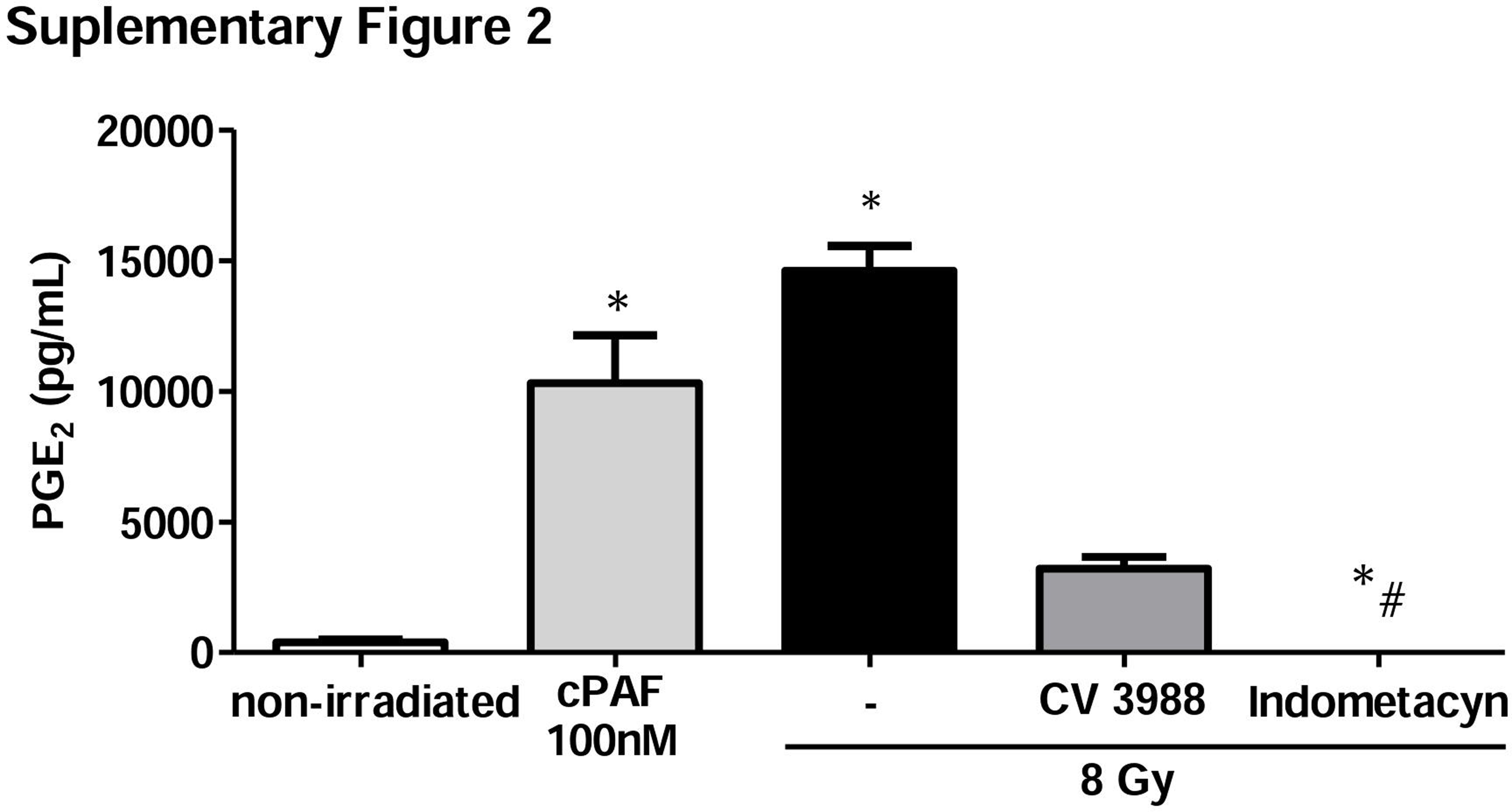

Supplement: Supplementary Figure 2 [file oncsis201690x3.tif]
